# 350. Joint Modeling of EHR and CXR Data to Predict COVID-19 Deterioration

**DOI:** 10.1093/ofid/ofab466.551

**Published:** 2021-12-04

**Authors:** Emily Mu, Sarah Jabbour, Michael Sjoding, John Guttag, Jenna Wiens, Adrian Dalca

**Affiliations:** 1 Massachusetts Institute of Technology, Naperville, IL; 2 University of Michigan, Ann Arbor, Michigan; 3 MIT, Cambridge, Massachusetts; 4 MIT, Harvard MGH, Cambridge, Massachusetts

## Abstract

**Background:**

Infectious respiratory-track pathogens are a common trigger of healthcare capacity strain, e.g. the COVID19 pandemic. Patient risk stratification models to identify low-risk patients can help improve patient care processes and allocate limited resources. Many existing deterioration indices are based entirely on structured data from the Electronic Health Record (EHR) and ignore important information from other data sources. However, chest radiographs have been demonstrated to be helpful in predicting the progress of respiratory diseases. We developed a joint EHR and chest x-ray (CXR) model method and applied it to identify low-risk COVID19+ patients within the first 48 hours of hospital admission.

**Methods:**

All COVID19+ patients admitted to a large urban hospital between March 2020 and February 2021 were included. We trained an image model using large public chest radiograph datasets and fine-tuned this model to predict acute dyspnea using a cohort from the same hospital. We then combined this image model with two existing EHR deterioration indices to predict the risk of a COVID19+ patient being intubated, receiving a nasal cannula, or being treated with a vasopressor. We evaluated models’ ability to identify low-risk patients by using the positive predictive value (PPV).

**Results:**

The image-augmented deterioration index was able to identify 12% of 716 COVID-19+ patients as low risk with 0.95 positive predictive value in the first 48 hours of admission. In contrast, when used individually, the EHR and CXR models each identified roughly 3% of the patients with a PPV of 0.95.

Predicting Low Risk Patients

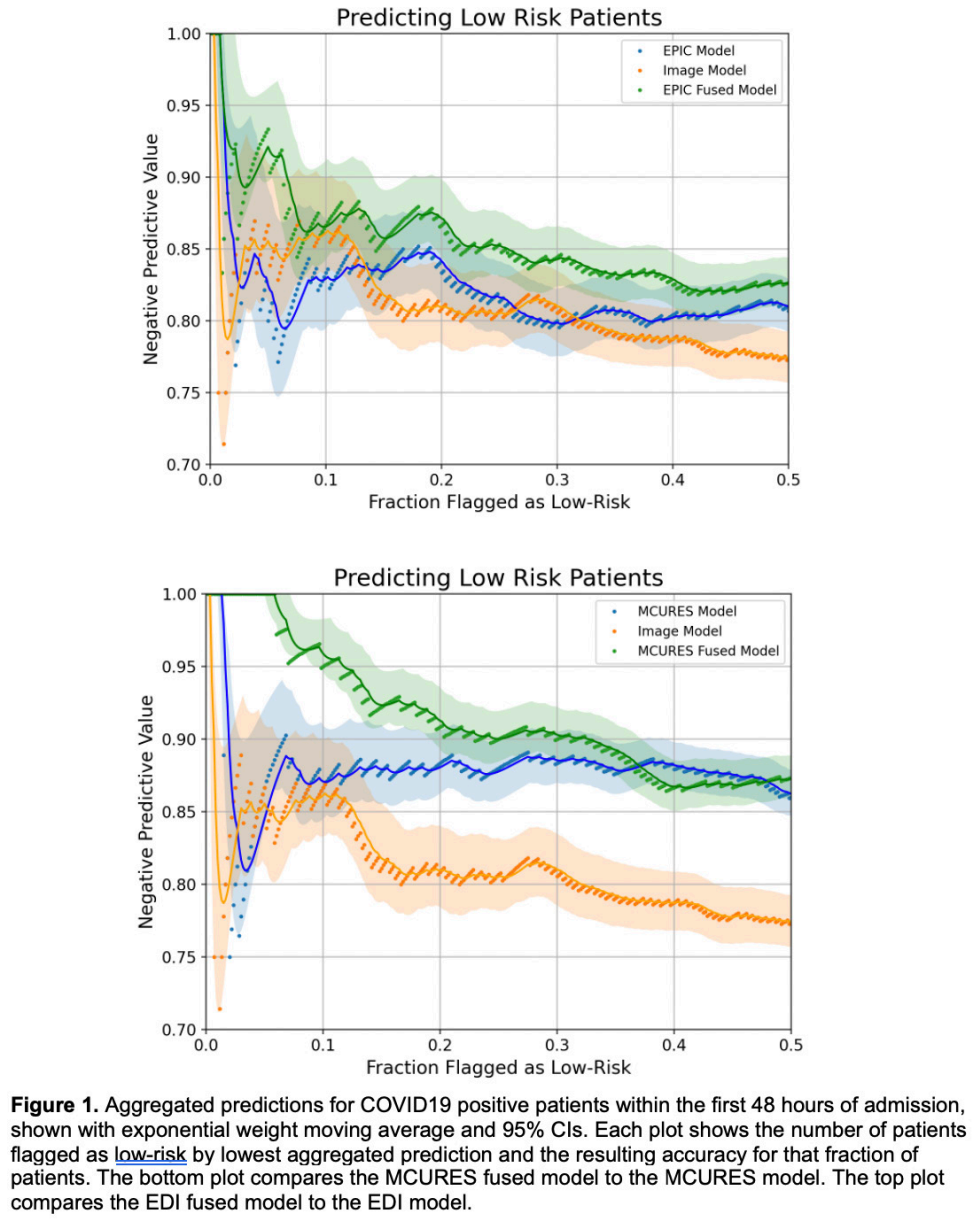

Aggregated predictions for COVID19 positive patients within the first 48 hours of admission, shown with exponential weight moving average and 95% CIs. Each plot shows the number of patients flagged as low-risk by lowest aggregated prediction and the resulting accuracy for that fraction of patients. The bottom plot compares the MCURES fused model to the MCURES model. The top plot compares the EDI fused model to the EDI model.

**Conclusion:**

Our multi-modal models were able to identify far more patients at low-risk of COVID19 deterioration than models trained on either modality alone. This indicates the importance of combining structured data with chest X-rays when creating a deterioration index performance for infectious respiratory-track diseases.

**Disclosures:**

**All Authors**: No reported disclosures

